# The effect of hospital care on early survival after penetrating trauma

**DOI:** 10.1186/s40621-014-0024-1

**Published:** 2014-09-17

**Authors:** David E Clark, Peter C Doolittle, Robert J Winchell, Rebecca A Betensky

**Affiliations:** 1 Department of Surgery, Maine Medical Center, 887 Congress Street, Suite 210, Portland, 04102 ME USA; 2 Center for Outcomes Research and Evaluation, Maine Medical Center, Portland, 04101 ME USA; 3 Tufts University School of Medicine, Boston, 02111 MA USA; 4 Harvard Injury Control Research Center, Harvard School of Public Health, Boston, 02115 MA USA; 5 Department of Biostatistics, Harvard School of Public Health, Boston, 02115 MA USA

**Keywords:** Trauma, Injury, Penetrating, Time-to-event analysis, Survival analysis, Regression, Interval censoring, Time-varying covariates, NTDB, NVDRS

## Abstract

**Background:**

The effectiveness of emergency medical interventions can be best evaluated using time-to-event statistical methods with time-varying covariates (TVC), but this approach is complicated by uncertainty about the actual times of death. We therefore sought to evaluate the effect of hospital intervention on mortality after penetrating trauma using a method that allowed for interval censoring of the precise times of death.

**Methods:**

Data on persons with penetrating trauma due to interpersonal assault were combined from the 2008 to 2010 National Trauma Data Bank (NTDB) and the 2004 to 2010 National Violent Death Reporting System (NVDRS). Cox and Weibull proportional hazards models for survival time (*t*_SURV_) were estimated, with TVC assumed to have constant effects for specified time intervals following hospital arrival. The Weibull model was repeated with *t*_SURV_ interval-censored to reflect uncertainty about the precise times of death, using an imputation method to accommodate interval censoring along with TVC.

**Results:**

All models showed that mortality was increased by older age, female sex, firearm mechanism, and injuries involving the head/neck or trunk. Uncensored models showed a paradoxical increase in mortality associated with the first hour in a hospital. The interval-censored model showed that mortality was markedly reduced after admission to a hospital, with a hazard ratio (HR) of 0.68 (95% CI 0.63, 0.73) during the first 30 min declining to a HR of 0.01 after 120 min. Admission to a verified level I trauma center (compared to other hospitals in the NTDB) was associated with a further reduction in mortality, with a HR of 0.93 (95% CI 0.82, 0.97).

**Conclusions:**

Time-to-event models with TVC and interval censoring can be used to estimate the effect of hospital care on early mortality after penetrating trauma or other acute medical conditions and could potentially be used for interhospital comparisons.

**Electronic supplementary material:**

The online version of this article (doi:10.1186/s40621-014-0024-1) contains supplementary material, which is available to authorized users.

## Background

Attempts to compare the effectiveness of different hospitals should consider time elapsed between injury and medical intervention and ideally should also account for subjects who die before reaching a hospital (Clark et al. [2012b]). Statistical methods, including Cox regression, have been developed for situations where the outcome of interest is the time from exposure (e.g., injury) until the occurrence of an event; since this event is usually death, these methods are often described as ‘survival analysis’ (Collett [1994]; Kalbfleisch and Prentice [2002]). Time-to-event methods (a more general term) can accommodate a variety of covariate effects, including ‘time-varying’ covariates (Dekker et al. [2008]; del Junco et al. [2013]), which are not present initially but begin at a specified time (e.g., hospital care). This study was undertaken to apply these methods to study victims of penetrating trauma and estimate the improvement in their survival associated with arrival at a hospital.

Unfortunately, implementation of time-to-event methods for medical emergencies is made more difficult because the time of death may be inconsistently recorded. The precise time may be unobserved if it occurs before Emergency Medical Services (EMS) personnel have arrived; conversely, declaration of death in the hospital (even for those ‘Dead on Arrival’ (DOA)) may be delayed until after resuscitative efforts have been judged futile. This inexactness creates a distribution of recorded survival times with an anomalous secondary mode (Clark et al. [2012a]). It may therefore be preferable to consider these early deaths as having occurred at some unspecified point during a defined interval, assuming only that the subject was alive at the beginning of the interval and dead at the end of the interval. Such ‘interval censoring’ can be incorporated within the framework of time-to-event analysis (Lindsey and Ryan [1998]; Zhang and Sun [2010]), but the combination of time-varying covariates (TVC) and an interval-censored outcome is not routinely handled by standard statistical software.

Clark et al. ([2013]) have recently described a method for the analysis of traffic crash mortality using TVC and interval censoring. The present study applies this method to subjects with penetrating trauma, utilizing two sources of data to estimate the time-varying effects of hospital intervention on mortality. This approach allows the relative effect of delay in reaching a hospital to be separated from other factors affecting the outcome and could be used to better compare one hospital or trauma system to another.

## Methods

This study was proposed to an Institutional Review Board, which ruled it exempt from further review because it uses existing deidentified records. Data management, modeling, and graphing were performed using Stata (Version 12, StataCorp, College Station, TX, USA), especially its time-to-event commands *stset*, *stsplit*, *stcox*, and *streg*.

### Data sources and data management

Data for patients with penetrating trauma for 2008 to 2010 were obtained from the National Trauma Data Bank (NTDB) of the American College of Surgeons (ACS), consistent with its Data Use Agreement (www.facs.org/trauma/ntdb). Only cases with an interpersonal mechanism of injury were included; firearm injuries were distinguished from other penetrating injuries. Indicator variables were created for wounds to the head/neck (AIS > 0 in the head or facial region) or trunk (AIS > 0 in the thorax, abdominal, or spine region). The verification level of the trauma center to which the patient was taken was noted (ACS level I versus other).

NTDB contains trauma registry information on the circumstances, treatments, and outcomes for each patient. Since 2008, this includes the time in minutes from the arrival of EMS personnel at the scene of the incident until the arrival of the patient at the hospital, which we designate *t*_PRE_. (NTDB does not contain information about the time prior to EMS arrival, which we assumed to be relatively brief in most cases.) NTDB does record the time in minutes from hospital arrival until declaration of death, which we designate *t*_INPT_. For patients who die, we defined *t*_SURV_ = *t*_PRE_ + *t*_INPT_. We excluded from analysis those patients who had a calculated Injury Severity Score (ISS) less than 9, whose injuries should not have been associated with any significant risk of mortality. We also excluded patients who were received in transfer from another hospital, those who did not have a recorded value of *t*_PRE_ (or who had a value of *t*_PRE_ recorded as greater than 480 min), and those who died but did not have a recorded value of *t*_INPT_.

To provide a population-based sample of subjects with similar injuries who did not survive to reach a hospital, we added data from the National Violent Death Reporting System (NVDRS) for 2004 to 2010, consistent with its Data Use Agreement (www.cdc.gov/violenceprevention/NVDRS/index.html). Only cases with a penetrating wound and an interpersonal mechanism (ICD-9 E-Code 960–969) were included; firearm injuries were distinguished from other penetrating injuries. Subjects who were recorded as having been treated in a hospital were excluded to avoid double counting. Indicator variables were created for wounds to the head/neck or trunk. Time from injury until death, as recorded in NVDRS, was designated as *t*_SURV_. Time of EMS intervention was not recorded by NVDRS.

Covariates for analysis were limited to those available in both datasets. NTDB did not contain specific information about the location of incidents or hospitals. Because NVDRS did not record any measure of injury severity, even minor injuries to the head/neck or trunk recorded in NTDB were included in the corresponding category. After similar variables had been developed for the records from both data sources, they were combined into a single analytic data set.

### Hazard regression modeling

Cox regression models are the most common way to estimate covariate effects on a time-to-event variable. They are based on the ‘hazard function’, that is, the probability that a subject who has not yet experienced the event will do so in the next instant. For this study, non-parametric and parametric regression models were constructed with survival time (*t*_SURV_) as the dependent variable.

A Cox model assumes some unspecified baseline hazard function *h*_0_(*t*), and a hazard function *h*(*t*;*x*) for each individual:

1 $$h\;\left(t;x\right)=h_{0}\left(t\right)g\left(x\right)\text{,}$$

where *g*(*x*), sometimes called a ‘scale parameter’, is usually independent of time and a function of covariates (*x*_1_ … *x*_*k*_). The logarithm of *g*(*x*) may be estimated by modeling a linear combination of covariates with coefficients (*a*_1_ … *a*_*k*_), that is,

2 $$\log\left(g\left(x\right)\right)=a_{1}x_{1}+\ldots +a_{k}x_{k}$$

so

3 $$g\left(x\right)=\exp\left(a_{1}x_{1}\right)\ldots \exp\left(a_{k}x_{k}\right)$$

Covariate terms thus have multiplicative or proportional effects on the hazard; if a covariate *x*_*j*_ is limited to values of 0 or 1 (as in this study), then exp(*a*_*j*_) may be interpreted as a hazard ratio (HR) estimating the effect of that covariate.

When there is a biologically plausible shape to the distribution of times, and especially when there are anomalies in the data, parametric models may be useful. In this case, it seemed reasonable to assume that the hazard was greatest immediately after injury and steadily decreased the longer a subject survived. The simplest parametric time-to-event model with this characteristic is based on the Weibull distribution (Collett [1994]; Kalbfleisch and Prentice [2002]) and specifies

4 $$h_{0}\;\left(t\right)=p\;t^{p-1}\exp\left(a_{0}\right)\text{;}$$

the scale parameter *g*(*x*) is estimated as described above. This specification of *h*(*t*;*x*) leads to a convenient expression for the survival function *S*(*t*;*x*), namely,

5 $$S\;\left(t;x\right)=\exp\left(-g\left(x\right)\exp\left(a_{0}\right)\;t^{p}\right)$$

The ‘shape parameter’ *p* may be any positive number. If *p* = 1, *h*_*0*_(*t*) is constant (the simple exponential model); if *p* < 1, the baseline hazard decreases with time.

### Introduction of time-varying covariates

Parametric or non-parametric Cox models can be extended to include ‘time-varying’ covariates (Dekker et al. [2008]), which might indicate changes in the status or characteristics of the subjects. In this study, TVC were created to designate periods of observation for each person, separated into sequential intervals *I*_*0*_ … *I*_*4*_, where *I*_*0*_ = (0, *t*_PRE_), *I*_*1*_ = (*t*_PRE_, *t*_PRE_ + 30), *I*_*2*_ = (*t*_PRE_ + 30, *t*_PRE_ + 60), *I*_*3*_ = (*t*_PRE_ + 60, *t*_PRE_ + 120), *I*_4_ = (*t*_PRE_ + 120, maximum observed time).

The covariate function *g*(*x*;*t*), now also a function of time, can therefore be fully specified by a linear combination

6 $$\begin{array}{c} \log\left(g\left(x;t\right)\right)=\;a_{1}x_{1}+\ldots +\;a_{k}x_{k}\\ +b_{1}I_{1}+b_{2}I_{2}+b_{3}I_{3}+b_{4}I_{4} \end{array}\text{,}$$

where the *a*'s, and *b*'s are the coefficients estimated from the data. Within each time interval, *g*(*x*;*t*) will be constant, specifically

7 $$\begin{array}{cc} \log\left(g_{0}\left(x;I\right)\right)=a_{1}x_{1}+\ldots +\;a_{k}x_{k} & \mathrm{for}\;t\;\mathrm{in}\;I_{0}\\ \log\left(g_{i}\left(x;I\right)\right)=\;\log\left(g_{0}\left(x;I\right)\right)+b_{i}I_{i} & \mathrm{for}\;t\;\mathrm{in}\;I_{1}\ldots I_{4} \end{array}$$

For the Weibull model, the survival function in each time interval can then be calculated as

8 $$\begin{array}{cc} S\left(t;x,I\right)=\exp\left(-g_{0}\left(x,I\right)\;\exp\left(a_{0}\right)\;t^{p}\right) & \mathrm{for}\;t\;\mathrm{in}\;I_{0}\\ S\left(t;x,I\right)=S\left(t_{\mathrm{PRE}}\right)\;\exp\left(-g_{1}\left(x,I\right)\;\exp\left(a_{0}\right)\;\left(t^{p}-\;{\left(t_{\mathrm{PRE}}\right)}^{p}\right)\right) & \mathrm{for}\;t\;\mathrm{in}\;I_{1}\\ S\left(t;x,I\right)=S\left(t_{\mathrm{PRE}}+30\right)\;\exp\left(-g_{2}\left(x,I\right)\;\exp\left(a_{0}\right)\;\left(t^{p}-\;{\left(t_{\mathrm{PRE}}+30\right)}^{p}\right)\right) & \mathrm{for}\;t\;\mathrm{in}\;I_{2}\\ S\left(t;x,I\right)=S\left(t_{\mathrm{PRE}}+60\right)\;\exp\left(-g_{3}\left(x,I\right)\;\exp\left(a_{0}\right)\;\left(t^{p}-\;{\left(t_{\mathrm{PRE}}+60\right)}^{p}\right)\right) & \mathrm{for}\;t\;\mathrm{in}\;I_{3}\\ S\left(t;x,I\right)=S\left(t_{\mathrm{PRE}}+120\right)\;\exp\left(-g_{4}\left(x,I\right)\;\exp\left(a_{0}\right)\;\left(t^{p}-\;{\left(t_{\mathrm{PRE}}+120\right)}^{p}\right)\right) & \mathrm{for}\;t\;\mathrm{in}\;I_{4} \end{array}$$

### Introduction of interval censoring

In the present study, non-parametric and Weibull models were estimated using *t*_SURV_ calculated first simply as the difference between the recorded injury time and declared death time. Another Weibull model was then estimated with the survival time for some subjects treated as ‘interval censored’, that is, to have occurred within a certain interval, as shown in Table 1. For persons who died without hospitalization, had no pulse at the time of hospital admission, or were classified DOA, the actual time of death was assumed to be somewhere between 1 min after injury and the declared time of death (*t*_SURV_). For other persons whose death was declared in the hospital, the actual time of death was assumed to be somewhere between the time of hospital arrival (*t*_PRE_) and the declared time of death (*t*_SURV_). The endpoints of these intervals may be designated *t*_LEFT_ and *t*_RIGHT_, conceiving of time as moving from left to right. A final adjustment in each case modified *t*_LEFT_ so that it was no earlier than 30 min prior to *t*_RIGHT_ (Table 1).

**Table 1:** **Left and right endpoints for interval censoring of survival times with different available data**

	Interval assumed to contain true death time	
	Left	Right
Died without hospital admission	Max(1, *t*_SURV_ − 30)	*t*_SURV_
Died in hospital, no pulse on admission	Max(1, *t*_SURV_ − 30)	*t*_SURV_
Died in hospital, pulse on admission	Max(*t*_PRE_, *t*_SURV_ − 30)	*t*_SURV_

*t*_PRE_, prehospital time; *t*_SURV_, declared death time.

An imputation/estimation algorithm (Clark et al. [2013]) was used to estimate the Weibull model with both TVC and an interval-censored survival time. The imputation/estimation algorithm was implemented as follows:

1. Select an arbitrary imputed survival time (*t*_IMP_) between *t*_LEFT_ and *t*_RIGHT_.
2. Estimate a Weibull model using standard Stata commands.
3. From the current model, determine *S*(*t*) for each subject, for all possible values of *t* in the interval from *t*_LEFT_ to *t*_RIGHT_*.*
4. Generate a uniform random number *u* between 0 and 1.
5. Replace *t*_IMP_ for each subject such that *S*(*t*_IMP_) is as close as possible to
  9 $$S\left(t_{\mathrm{LEFT}}\right)-u*\left(S\left(t_{\mathrm{LEFT}}\right)-S\left(t_{\mathrm{RIGHT}}\right)\right)$$
6. Estimate a Weibull model again.
7. Stop if model coefficients are stable to two decimal places; otherwise, return to step 3.

## Results

For 2008 to 2010, NTDB contained records for 73,916 patients who had an ISS of at least 9 and were received directly by the reporting hospital after penetrating injuries, of which 58,399 (79.0%) had resulted from interpersonal violence. Among NTDB patients with penetrating trauma due to interpersonal violence, only 29,008 (51.2%) had valid and non-missing data for outcome (lived/died), hospitalization status, time to death (*t*_SURV_), and time to hospital arrival (*t*_HOS_); ISS of 16 to 24 was recorded for 5,602 patients (19.2%) and ISS of 25 to 75 was recorded for 5,081 patients (17.5%).

For 2004 to 2010, NVDRS contained records for 64,936 persons in 18 states who died after penetrating trauma, of whom 24,619 (37.9%) died as a result of interpersonal violence; 13,955 (56.7%) of the latter group died without transportation to a hospital. Of the non-hospital deaths, 3,473 (24.9%) were female, but of the hospital deaths, only 1,195 (11.2%) were female. Only the non-hospitalized NVDRS subjects were included in the subsequent analysis; among these, only 4,824 (34.6%) had valid and non-missing data for outcome and time to death; NVDRS contained only fatal cases and did not record any injury severity scores.

Selected characteristics of subjects from NTDB, NVDRS, and the combined sample are shown in Table 2. Characteristics of cases with valid and non-missing data are also shown. About 27.7% of the subjects in the final analysis sample died, including 7,308 (21.6%) in the first 240 min.

**Table 2:** **Characteristics of subjects from NTDB, NVDRS, and the combined sample**

	NTDB	NVDRS	Combined
Cases not excluded	58,399	13,955	72,354
Age 0 to 39	48,065 (82.3%)	9,257 (66.3%)	57,322 (79.2%)
Age 40 to 64	9,813 (16.8%)	3,972 (28.5%)	13,785 (19.1%)
Age 65-79	452 (0.8%)	538 (3.9%)	990 (1.4%)
Age 80 or more	69 (0.1%)	163 (1.2%)	232 (0.3%)
Male	52,698 (90.3%)	10,482 (75.1%)	63,180 (87.3%)
Female	5,526 (9.5%)	3,473 (24.9%)	8,999 (12.4%)
Firearm mechanism	38,994 (66.8%)	11,322 (81.1%)	50,316 (69.5%)
Non-firearm mechanism	19,405 (33.2%)	2,633 (18.9%)	22,308 (30.5%)
Head/neck injury	15,659 (26.8%)	7,743 (55.5%)	23,402 (32.3%)
Trunk injury	43,218 (74.0%)	7,630 (54.7%)	50,848 (70.3%)
No head/neck/trunk injury	8,690 (14.9%)	1,690 (12.1%)	10,380 (14.4%)
Level I trauma center	25,534 (43.7%)	0	25,534 (35.3%)
Died	8,540 (14.7%)	13,955 (100%)	22,495 (31.1%)
Cases included with valid/non-missing data	29,008	4,824	33,832
Age 0 to 39	23,426 (80.8%)	3,277 (67.9%)	26,703 (78.9%)
Age 40 to 64	5,307 (18.3%)	1,335 (27.7%)	6,642 (19.6%)
Age 65 to 79	242 (0.8%)	164 (3.4%)	406 (1.2%)
Age 80 or more	33 (0.1%)	46 (1.0%)	79 (0.2%)
Male	26,088 (90.0%)	3,651 (75.7%)	29,739 (87.9%)
Female	2,915 (10.1%)	1,173 (24.3%)	4,088 (12.1%)
Firearm mechanism	19,755 (68.1%)	4,051 (84.0%)	23,806 (70.4%)
Non-firearm mechanism	9,253 (31.9%)	773 (16.0%)	10,026 (29.6%)
Head/neck injury	7,753 (26.7%)	2,524 (52.3%)	10,277 (30.4%)
Trunk injury	21,854 (75.3%)	2,607 (54.0%)	24,461 (72.3%)
No head/neck/trunk injury	4,178 (14.4%)	693 (14.4%)	4,871 (14.4%)
Level I trauma center	12,450 (42.9%)	0	12,450 (36.8%)
Died	4,547 (15.7%)	4,824 (100%)	9,371 (27.7%)
Died < 240 min	3,725 (12.8%)	3,583 (74.3%)	7,308 (21.6%)

Cases with Injury Severity Score less than 9 or received in transfer from another hospital were excluded from NTDB data. Cases admitted to a hospital were excluded from NVDRS data.

Non-parametric and Weibull models were estimated, first with recorded survival time (*t*_SURV_) as an outcome (Table 3). Increased mortality was associated in these models with older age, female sex, firearms, and wounds of the head/neck or trunk. The apparent effect of female sex was greater with older age. These uncensored models do not adjust for the artificial second peak in survival times resulting from delayed declaration of death and accordingly associated initial hospital intervention with an increased hazard. No effect of level I trauma center status was apparent.

**Table 3:** **Results obtained from regression models**

	Cox model		Weibull model		Weibull model	
	Uncensored		Uncensored		Interval-censored	
	HR	95% CI	HR	95% CI	HR	95% CI
Male age 40 to 64 vs male age 0 to 39	1.27	1.21, 1.34	1.28	1.21, 1.35	1.21	1.14, 1.28
Male age 65 to 79 vs male age 0 to 39	1.66	1.41, 1.96	1.82	1.52, 2.18	1.53	1.25, 1.86
Male age ≥80 vs male age 0 to 39	1.81	1.24, 2.64	2.04	1.40, 2.99	1.63	1.06, 2.51
Female age 0 to 39 vs male age 0 to 39	1.34	1.26,1.43	1.44	1.35, 1.54	1.35	1.26, 1.45
Female age 40 to 64 vs male age 40 to 64	1.39	1.26, 1.53	1.58	1.42, 1.75	1.44	1.28, 1.61
Female age 65 to 79 vs male age 65 to 79	1.78	1.32, 2.43	1.87	1.36, 2.56	1.84	1.29, 2.61
Female age ≥80 vs male age ≥80	1.29	0.73, 2.30	0.97	0.44, 2.14	0.78	0.33, 1.88
Firearm vs other mechanism	2.89	2.73, 3.06	2.99	2.82, 3.16	2.94	2.77, 3.12
Head/neck injury vs none	2.07	1.99, 2.16	2.21	2.12, 2.30	2.09	2.00, 2.18
Trunk injury vs none	1.24	1.19, 1.29	1.23	1.18, 1.29	1.30	1.24, 1.37
Hospital 0 to 30 min vs prehospital	3.20	2.92, 3.49	2.35	2.22, 2.49	0.68	0.63, 0.73
Hospital 31 to 60 min vs prehospital	0.89	0.76, 1.03	0.55	0.49, 0.61	0.22	0.19, 0.25
Hospital 61 to 120 min vs prehospital	0.46	0.39, 0.54	0.42	0.38, 0.47	0.29	0.26, 0.32
Hospital >120 min vs prehospital	0.06	0.05, 0.06	0.023	0.020, 0.025	0.012	0.011, 0.013
Level I trauma center vs other hospital	1.00	0.94, 1.06	1.00	0.94, 1.06	0.93	0.82, 0.97
Constant			0.0070	0.0064, 0.0076	0.0089	0.0082, 0.0097
Shape parameter			0.42	0.41, 0.43	0.45	0.44, 0.46

For Weibull models, the constant multiplied by the applicable hazard ratios gives the scale parameter *g*(*x*;*t*); Weibull models also have a shape parameter *p*, as described in the text. HR, hazard ratio; CI, confidence interval.

**Figure 1:**
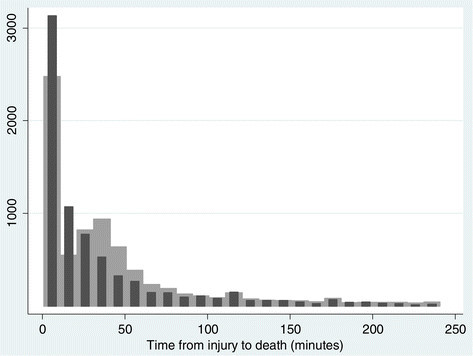
**Distribution of death times.** Distribution of recorded death times (*t*_SURV_) in the sample (lighter grey histogram) and distribution of imputed death times (*t*_IMP_) at the conclusion of the imputation/estimation algorithm described in the text (darker grey bars).

A Weibull model was then estimated with the assumption of interval censoring, imputing a death time (*t*_IMP_) according to the imputation/estimation algorithm described earlier. The smoothed distribution of *t*_IMP_ is compared to the bimodal distribution of *t*_SURV_ in Figure 1. The results of the interval-censored Weibull model were consistent regardless of the initial value selected for *t*_IMP_ and converged after a few iterations to the values shown in the last column of Table 3. In the interval-censored model, increased mortality was again associated with older age, female sex, firearms, and injuries to the head, neck, or trunk. The apparent effect of female sex was again greater with older age. Hospital intervention was associated with a large reduction in the hazard, and this association became stronger the longer a subject was in the hospital. If the hospital was a level I trauma center, the hazard was further decreased.

**Figure 2:**
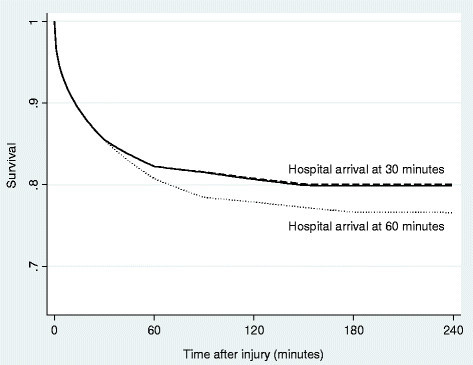
**Predicted survival curves.** The solid line depicts the survival curve predicted from the interval-censored Weibull model for a 30-year-old man with a gunshot wound to the trunk, who arrives at a hospital that is not a level I trauma center 30 min after injury. The dashed line depicts the improved survival for a similar man who arrives at a level I trauma center 30 min after injury. The dotted line depicts the decreased survival for a similar man who arrives at a level I trauma center 60 min after injury.

Hypothetical survival curves for individual subjects are plotted in Figure 2. These contrast the differences in expected survival for 30-year-old men with a gunshot wound to the trunk (chest or abdomen), depending upon when they arrived at a hospital and whether the hospital was a level I trauma center. It appears that the benefit of arriving at a hospital with greater resources may be offset if the prehospital time is prolonged beyond a certain point.

## Discussion

Evaluating the effect of emergency medical care is challenging because of the constraints of geography and resources, rapidly changing risks and covariates, and limitations in the available data. Outcomes may be affected by variability in the timing of interventions, in addition to variability in the quality of interventions. Time-to-event statistical methods with TVC are therefore appropriate when comparing populations and treatments for acute conditions.

A previous study of early mortality in trauma centers (excluding patients pulseless on admission) demonstrated that the baseline hazard decreased steadily after the first 15 min of arrival and that the overall hazard was increased by age, physiologic derangements, injury severity, and firearm injury mechanism (Clark et al. [2012b]). Hospital-specific effects varied significantly depending on the time elapsed since injury. The authors emphasized that a more comprehensive system-based evaluation would require extending the hazard modeling approach to include prehospital data.

Studies of early survival after trauma are especially subject to bias because the ‘left truncation’ of injured persons who die before intervention is possible. Prehospital deaths are usually not included in clinical databases, and if the analysis does not properly account for this omission, inferences from an observational study may be affected by ‘survival bias’ (del Junco et al. [2013]). In particular, a study excluding patients who die without being transported by EMS may not allow valid inferences about the effect of prehospital time on outcome (Newgard et al. [2010]).

Analysis of early mortality after trauma is further complicated by difficulty specifying the time of death. For most subjects not in a hospital, the ‘time of death’ probably refers to the conventional definition of death as circulatory arrest, but may not have been actually witnessed. For most subjects in a hospital, the ‘time of death’ generally refers to the cessation of resuscitative efforts attempting to reverse a circulatory arrest that occurred some time earlier. Interval censoring offers a method to incorporate these inconsistencies into a mathematical model (Clark et al. [2012a]).

The Weibull model is one of the simplest parametric time-to-event models but is only one of the several that could be used with an interval-censored outcome. A non-parametric Cox model entails fewer assumptions, but parametric models can incorporate the reasonable assumption of a decreasing hazard, especially when data are sparse or inconsistent. ‘Piecewise’ parametric models that break the analysis into two or more time intervals allow almost as much flexibility as a non-parametric model (Friedman [1982]). Another advantage of parametric models (including piecewise parametric models) is that survival or hazard functions can be expressed as mathematical formulas for calculations or graphing.

Standard software packages (or supplements contributed by their user communities) provide methods for estimation of interval-censored time-to-event models (Griffin [2005]) or for models with TVC. However, estimating models with both an interval-censored outcome and TVC is more difficult (Clark et al. [2013]). The ‘imputation/estimation algorithm’ described above is a relatively simple and effective solution to this difficulty.

No contemporary database is available that includes EMS arrival times, hospital arrival times, and death times for a population-based sample of assault victims including those not transported to a hospital. Even the promising National Emergency Medical Services Information System (NEMSIS) excludes patients not transported by EMS and lacks outcome information after hospital arrival for most cases. Some combination of databases with compatible information is therefore necessary for a population-based study of these injuries, and our combination of NTDB and NVDRS demonstrates how this may be accomplished.

It was not surprising to find an association of older age, firearm mechanism, and injury to the head/neck or chest with increased mortality due to penetrating injuries resulting from interpersonal violence. However, the association of female sex with increased mortality is noteworthy, especially since some studies of hospitalized trauma patients have demonstrated an association of male sex with increased mortality (Haider et al. [2010]; Petersen et al. [2013]). NVDRS data show that women comprise only 11.2% of the hospital deaths, but 24.9% of the non-hospital deaths, and more detailed analysis of this disparity is certainly warranted. In the meantime, investigators asserting a survival advantage for injured females should be cautious when making inferences based on observational data limited to hospitalized subjects.

The findings in the present study are also generally consistent with the clinical intuition and assumptions underlying trauma systems. They provide some quantification of the relative magnitude of the effects of hospital intervention and trauma center status, which can be difficult to disentangle (Kidher et al. [2012]; Frezza and Mezghebe [1999]). The effect of delay clearly increases with time, validating the basic concept of a ‘golden hour’ (Cowley [1976]; Cowley and Scanlan [1979]). In general, arrival at a hospital seems to be an important determinant of outcome, and if data were available for a specific region, it would be possible to predict for a specific subject whether transportation to a more specialized trauma center would justify any additional delay.

### Limitations

NTDB and NVDRS each have complete data for only a minority of cases. If these data are not missing at random, conclusions based on complete case analysis may be biased (del Junco et al. [2013]). Since our purpose was to demonstrate methods of survival analysis, we chose not to complicate the analysis by imputation of variables other than survival time. NTDB does not include information about time elapsed prior to EMS arrival, which we had to assume was relatively brief. NTDB also does not identify the location of incidents or hospitals, so we were not able to extend the present analysis to include regional or even rural/urban effects. NVDRS does not contain any measure of injury severity or information about injury victims who did not die, and does not include the times of EMS or hospital intervention.

NTDB only contains data from hospitals that maintain a trauma registry, which generally limits it to trauma centers or similar hospitals with a special commitment to the care of injured patients; we do not know whether the findings of this study can be generalized to other hospitals. NVDRS does not have the clinical detail found in a trauma registry and only contains data from the minority of states that participate in this program, so we also do not know whether our findings would be the same with more detailed data or whether they can be generalized to other states.

Our combination of two data sets with similar but not identical time periods and locations should temper any conclusions based upon the models presented in this report. However, in the absence of any single data set that includes equivalent data for both hospitalized and non-hospitalized patients, this combination may be the best available approximation. Anticipating future maturation and even merger of NEMSIS and NTDB, however, the methods described here may be increasingly useful for the evaluation of trauma centers and trauma systems.

## Conclusions

Time-to-event analysis may be useful for understanding the effects of trauma systems and emergency care in general. These methods could be applied in other disease processes where medical care is time-limited, for example, acute myocardial infarction or stroke (Heestermans et al. [2010]; Fassbender et al. [2013]). We have specifically demonstrated that uncertain event times can be incorporated into such models using interval censoring and are not necessarily a barrier to the implementation of TVC indicating hospital arrival or other interventions occurring before or after hospital arrival.

## Authors’ original submitted files for images

Below are the links to the authors’ original submitted files for images. Authors’ original file for figure 1 Authors’ original file for figure 2
