# Supplementary figures and images for: The effect of hospital care on early survival after penetrating trauma
Source: Inj Epidemiol. 2014 Sep 17;1(1):24. doi: 10.1186/s40621-014-0024-1 (PMC5005558; doi:10.1186/s40621-014-0024-1)

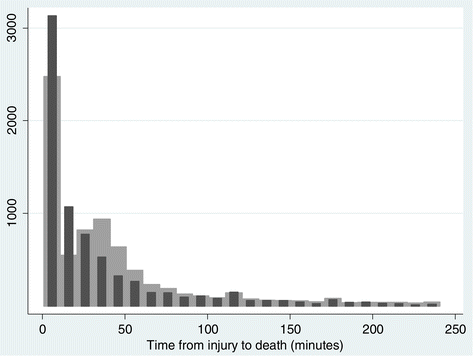

Supplement: Supplementary file 1 — Authors’ original file for figure 1 [file 40621_2014_24_MOESM1_ESM.gif]

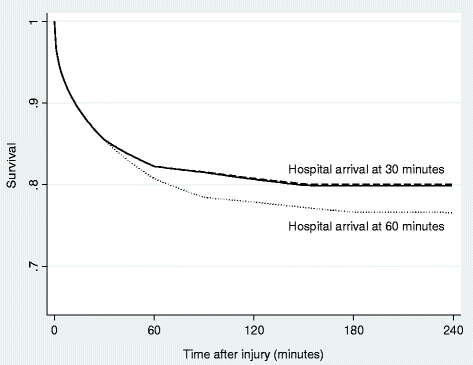

Supplement: Supplementary file 2 — Authors’ original file for figure 2 [file 40621_2014_24_MOESM2_ESM.gif]
